# Global positioning system technology (GPS) for psychological research: a test of convergent and nomological validity

**DOI:** 10.3389/fpsyg.2013.00315

**Published:** 2013-06-03

**Authors:** Pedro S. A. Wolf, Aurelio J. Figueredo, W. Jake Jacobs

**Affiliations:** ^1^Department of Psychology, The University of Cape TownCape Town, South Africa; ^2^Department of Psychology, The University of ArizonaTucson, AZ, USA

**Keywords:** global positioning system, physical activity, individual differences, convergent validity, nomological validity

## Abstract

The purpose of this paper is to examine the convergent and nomological validity of a GPS-based measure of daily activity, operationalized as Number of Places Visited (NPV). Relations among the GPS-based measure and two self-report measures of NPV, as well as relations among NPV and two factors made up of self-reported individual differences were examined. The first factor was composed of variables related to an Active Lifestyle (AL) (e.g., positive affect, extraversion…) and the second factor was composed of variables related to a Sedentary Lifestyle (SL) (e.g., depression, neuroticism…). NPV was measured over 4 days. This timeframe was made up of two week and two weekend days. A bi-variate analysis established one level of convergent validity and a Split-Plot GLM examined convergent validity, nomological validity, and alternative hypotheses related to constraints on activity throughout the week simultaneously. The first analysis revealed significant correlations among NPV measures- weekday, weekend, and the entire 4-day time period, supporting the convergent validity of the Diary-, Google Maps-, and GPS-NPV measures. Results from the second analysis, indicating non-significant mean differences in NPV regardless of method, also support this conclusion. We also found that AL is a statistically significant predictor of NPV no matter how NPV was measured. We did not find a statically significant relation among NPV and SL. These results permit us to infer that the GPS-based NPV measure has convergent and nomological validity.

## Global positioning system technology (GPS) for psychological research: a test of convergent and nomological validity

The primary purpose of this paper is to determine the convergent validity (Cronbach and Meehl, 1955; Campbell, 1960) of a GPS-based measure of daily activity in humans. Determining the psychometric properties of direct real-time behavior as they occur is a first step in ensuring that such measures adequately represent the constructs being measured. Most measures available to psychologists are either self-report or prohibitively expensive both in terms of time and money. To do so, we examined the relations among the GPS-based measure and two self-report measures of the same construct.

The second purpose of this paper is to examine a number of nuanced accounts of these data that suggest our design did not provide a clear demonstration of convergence among the measures of daily activity. The third and final purpose is to determine the nomological validity of the GPS-based measure in reference to a nomological net consisting of two additional terms that included six subjective individual differences measures which relate to daily activity and the two self-report measures of daily activity.

The face validity of the primary measure in this study, self-reported and GPS-based recorded Number of Places Visited (NPV), is obvious. The self-report measure provides an aggregate measure of the level of daily activity in geographic space and the GPS-based measure provides a moment-by-moment measure of daily activity in the same space. Both approaches appear to measure what they are supposed to measure, NPV, their purpose is obvious, and both use the same metric. Hence, data obtained from the approaches are directly comparable. This shared metric permitted us to examine two forms of construct validity, convergent validity and nomological validity directly.

### Convergent validity

Convergent validity is a form of construct validity. It is the degree that different methods of measuring the same construct agree (Campbell and Fiske, 1959). To examine convergent validity we examined correlations among a Diary-based measure of Number of Places Visited(Diary-NPV), a GoogleMaps based measure of Number of Places Visited (Google-NPV), and a GPS-based measure of Number of Places Visited (GPS-NPV) obtained from our participants over 4 days (96 consecutive hours). If the GPS-based measure of NPV converges with the Diary-NPV and the Google-NPV, we will see positive correlations among the GPS- and self-report-based measures (see e.g., Cronbach and Meehl, 1955).

Because each participant provided data from two weekdays and a full weekend, we could directly compare activity on weekdays and weekends, accomplishing a tertiary purpose. Perhaps participants are familiar with a stable weekday schedule and, as a result, accurately self-report NPV for weekdays, but, because they are less familiar with their unscheduled weekends, their weekend self-report accuracy suffers. If this is true, we will observe higher correlations among the measures of NPV on the weekdays than on the weekends. This is of course only one of many possibilities. For example, perhaps participants systematically forget to carry the GPS devices on weekdays or do not have internet access on the weekends. These and other possibilities point to differences in the correlations among the NPV measures obtained during weekdays and weekends. To examine these possibilities, we compared the correlations obtained during the full 4 days, the weekend, and the weekday block. If these correlations remain stable, the data will have disconfirmed the full set of possibilities outlined above.

To more formally test these possibilities, we examined a number of interpretations of these data that suggest that convergent validity of these measures change as a function of circumstance. The principles illustrated by the example in preceding paragraph would produce a statistical interaction among the measures obtained by the Diary-, Google Maps- and GPS-based measures of NPV and the days on which the data were collected. Under these circumstances the mean GPS NPV will be higher than both the mean Google NPV, and mean Diary NPV. To test hypotheses such as these, we included interaction terms in two of the analyses.

Even if these data correlate perfectly, it remains possible that there is method-specific under or over reporting of NPV from each method. As a safeguard against this possibility, we compared the mean NPV obtained from the Diary, Google, and GPS methods across the 4 days. If we find no detectable differences among these means, then we have a second line of evidence supporting the convergent validity of the GPS-based measure and data inconsistent with the under or over reporting hypothesis.

### Nomological validity

Nomological validity is another form of construct validity. It is “… the degree to which a construct behaves as it should within a system of related constructs called a nomological net.” Campbell (1960); Campbell (p. 547). Campbell further stated that “… *nomological validity…* represent[s] the possibility of validating tests by using the scores from a test as interpretations of a certain term in a formal theoretical network and, through this to generate predictions which would be validating if confirmed when interpreted as still other operations and scores.”

**Figure 1:**
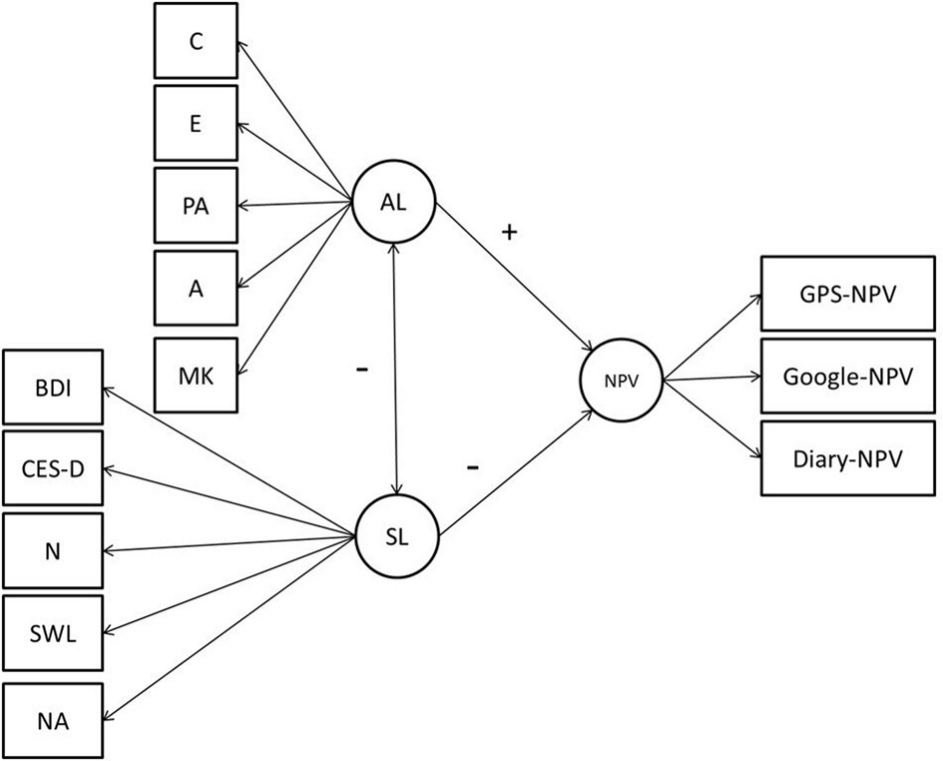
**Conceptual diagram of the nomological net**.

In our case, the literature is full of self-reported individual differences variables that relate to level of daily activity (e.g., depression, affect, personality) (e.g., Byrne and Byrne, 1993; Schwedfeger et al., 2010). Some of the variables (e.g., openness to experience, extraversion, positive affect), are related to an active lifestyle (e.g., Rhodes and Smith, 2006; Mata et al., 2012) and other variables (e.g., depression, negative affect, neuroticism), are related to a sedentary lifestyle (Rhodes and Smith, 2006; Wichers et al., 2012). Because the literature indicates positive relations with active lifestyles and a group of variables and positive relations with sedentary lifestyles and another group of variables, we predicted positive relations among those that predict an active lifestyle and among those that predict a sedentary lifestyle. This pattern of relations is one part of the nomological net permitting us to test an objective measure of active or sedentary lifestyles against a net constructed of self-report measures of individual differences. See Figure 1 for a visual representation of the nomological net.

To examine this proposed nomological net we constructed two unit weighted factors, the first measured variables known to predict an Active Lifestyle (AL), and the second measured variables known to predict a Sedentary Lifestyle (SL) and correlated those factors against the two self-reported NPV measures and our GPS measure hence testing the nomological validity of our NPV measures. We chose this technique rather than a factor analysis due to the small sample size of our intensive (relatively few participants with many measures) rather than extensive research design (many participants with few data points) (Kraemer, 1978), or reporting thirty individual bivariate correlations among our construct indicators in order to test hypotheses within the framework of more advanced GLM techniques.

In addition to the relationship between our individual AL and SL indicator variables and physical activity, the indicator variables comprising both of the theoretically specified factors were based on either prior factor analytic research (in the case of AL), or on empirical and theoretical relations among the indicators or constructs (in the case of SL). The indicator variables that went into constructing the AL factor (i.e., PANAS Positive Affect Scale, Mini-K Short Form, Conscientiousness, Extraversion, and Agreeableness) are all indicators of a higher order slow life history factor. The theoretical underpinnings of this factor are based in evolutionary life history theory and a full rationale would require an extensive discussion of that theoretical construct that is not directly related to daily physical activity and the scope of this methodological paper. For an introduction to the theoretical rationale and factor structure of these variables see Figueredo et al. (2004, 2007).

The indicators that compose the SL factor included two measures of depression (Beck Depresion Inventory (BDI) and [Center for Epidemiological Studies- Depression Scale (CESD)], a measure of neuroticism [the Neuroticism Scale from Neuroticism-Extroversion-Openness Inventory- Five Factor Inventory (NEO-FFI)], negative affect [Negative Affect Scale of the Positive Affect Negative Affect Scale (PANAS)] and a measure of total life satisfaction (inversely scored Satisfaction with Life Scale). Watson and Clark (1984) have characterized many of these diverse constructs as part of a stable trait. From this point of view, people who score highly on this trait tend to experience discomfort and dwell on negative aspects of life regardless of situation and in the absence of stress (Watson and Clark, 1984). This would include people who score high in neuroticism, depression, and negative affect. Unfortunately, studies that investigate the factor structure of all the variables, that we chose, are rare. One study conducted a Confirmatory Factor Analysis (CFA) investigating the factor structure of Life Satisfaction, Depression, Personality, Anxiety, and Positive Affect and measured Neuroticism (Headey et al., 1993).

The results of their CFA found that the Life Satisfaction and Depression factors are separable factors but that they are highly correlated with each other (−0.61). They found that the Positive Affect Factor, that is part of our AL factor, was relatively distinct in that there were only moderate relationships between Life Satisfaction, and Positive Affect (0.34) and Depression and Positive Affect (−0.26). Although the researchers also measured personality variables including Neuroticism and Negative Affect they did not include these variables in the confirmatory factor analysis, but instead correlated the factor scores, based on their CFA, with Neuroticism and found a moderate correlation between their Life Satisfaction factor and Neuroticism (0.33) and a more substantial correlation between Neuroticism and their Depression factor (0.46). Unfortunately, they did not report correlations between Negative Affect and their factors, but instead only reported bivariate correlations among the indicators. Negative Affect was positively correlated the BDI (0.42), the SWL scale (−0.40) and the correlation between Negative Affect and Neuroticism was not reported.

Theoretically and empirically, all of these variables appear to be part of this underlying trait that is linked to activity. If we are to demonstrate the nomological validity of our GPS-based NPV measure (GPS-NPV), we expect to see positive relations among AL, the two self-reported NPV measures and GPS-NPV and negative relations among SL, our two self-reported NPV measures, and our GPS-NPV. These relations should hold whether the activity data come from diaries, questionnaires, or interviews.

It is reasonable to argue, that, for many, work schedules influence daily activity, leveling individual differences of interest here. For example, individuals in our sample may be forced to go to and remain in a single place (or several places) during an eight hour workday, independent of AL or SL. On the weekends, however, unconstrained by a work schedule, individuals high on AL may visit more places than during the week whereas individuals high on SL may visit fewer.

To test this possibility, we examined relations among each of our NPV measures and the unit weighted factor representing SL and AL on weekend and on weekdays. If the present hypothesis is true, then AL and NPV will positively correlate and SL and NPV will negatively correlate on the weekends, but not on the weekdays. In the analysis, this will manifest as a significant interaction between a weekday/weekend variable and AL *and* as a significant interaction between the weekday/weekend variable and SL.

In other words, the regression lines relating NPV to AL and the regression line relating NPV to SL will be flat and indistinguishable on the weekdays, but on the weekend, the regression line describing the relation between weekend NPV and AL will be positive but the regression line describing relation between NPV and SL will be negative.

We used a bi-variate approach to establish one level of convergent validity. Following that, we used Split-Plot General Linear Models that permitted us to examine convergent validity, nomological validity, and alternative hypotheses related to constraints on activity throughout the week simultaneously.

## Materials and methods

### Participants

Ninety-six participants, 41 females and 46 males, and 9 others who declined to answer the ‘sex’ question in the demographics questionnaire, were recruited from the University of Arizona Psychology 101 subject pool. On average, the participants were 19 years of age (*SD* = 2.54); their ages ranged from 18 to 39 years of age. There were 51 Freshman, 21, Sophomores, 10 Juniors, and 5 Seniors.

Upon sign up, participants made three appointments, the first to receive the GPS units and instructions, a second to change the batteries in the GPS units, and a third to return the GPS unit, complete a Google NPV task (described below), and receive credit for participating in the study. We aimed for 48 of the participants to start on a Wednesday and the remaining 48 to start on a Friday. Unfortunately, the participants disproportionately signed up for the Wednesday time slots over the Friday timeslots. In the end, 67 participants began the study on a Wednesday and 29 participants began on a Friday.

## Materials and measures

### Diary NPV website

We created a website that required the participants to answer seven questions. For present purposes we used the data obtained from the following question:

How many different places did you visit today?

If you visited the same building more than once at different times, count it as many times as you visited it. For example, if you left your home in the morning and returned at night, count the home location twice.

Software automatically emailed a copy of the following reminder to each participant at the end of each *full* day they carried the GPS devices.

We have already asked you to complete four short questionnaires, one for each of the full days you will be carrying around the GPS device. This email is a reminder that today is one of those 4 days. The website for this study is https://xxxxxxxx.edu. On this website there are four links, one for each full day you are carrying the GPS device. Please fill out the questionnaire which corresponds to today. You will need your UA Net ID and password to log on to this site.

Thank you for participating in this study.

Hence, the Diary NPV website asked the participants to report the number of times they visited unique locations during that day. This procedure resembles the Day Reconstruction Method (Kahneman et al., 2004). By limiting recall to that day, this method reduces recall bias (Stone et al., 2006).

### Google NPV

We designed a “Google Maps task” as a second measure of NPV. The participants completed the Google Maps task on the last day of the study (Appointment 3, see below).

In this task, the participants recreated their movement from place to place on a Google Map (using www.maps.google.com). The participants were asked to think of their place-to-place movements on each day as a series of events, beginning with the place they slept that night. The participant located the first place (typically their home) on Google Maps. They then completed the Google Maps “ask for directions form,” specifying the initial place as the origin and the next place they visited as the destination. The participant repeated this procedure using the previous destination as a place of origin and the next place visited as a destination until all places they remembered visiting for Day 1 were recorded.

After completing this procedure for Day 1, the participants were presented with a new Google Map and repeated the procedure for Day 2, received a fresh Google Map and repeated the procedure for Day 3, and repeated the procedure for Day 4. The participants thereby systematically recreated the number and location of places they remembered visiting during the previous 4 days in the order that they remembered the visits happened.

### GPS NPV

The participants were asked to carry a LandSeaAir Tracking Key that collected longitude, latitude, time, altitude, speed, direction of travel once a second as long as the devices detected movement and could detect the necessary radio signals from GPS satellites. The participants carried these devices as described in the Procedure section.

### Individual differences website

We created a website that permitted the participants to complete five questionnaires: the Beck Depression Inventory (BDI), the Center for Epidemiological Studies- Depression Scale (CESD), the Positive Affect Negative Affect Scale (PANAS), the Satisfaction with Life Survey (SWL), the Mini-K (MK), and the Neuroticism-Extroversion-Openness Inventory- Five Factor Inventory (NEO-FFI).

#### Beck depression inventory (BDI)

The BDI-II is a 21-item multiple-choice self-report inventory composed of items relating to signs and symptoms of depression. Increasing scores indicate increasing levels of depression. Traditionally, scores ranging between 30 and 63 indicate severe depression, between 19 and 29 indicate moderate to severe, between 10 and 18 indicate mild depression, and scores ranging between 0 and 9 indicate not depressed (Beck and Beamesderfer, 1974).

Beck et al. (1988), who meta-analyzed 25 years of research, reported a mean internal consistency estimate of α = 0.87 and test-retest reliability greater than *r* = 0.60. The concurrent validity of the BDI with respect to other measures of depression ranges from *r* = 0.41 on the Zung to *r* = 0.81 on the MMPI-D.

#### Center for epidemiological studies- depression scale (CESD)

The CES-D is a 20-item screening test for depression. This instrument has a high internal consistency (α = 0.85 in the general population and α = 0.90 in a patient sample) and test-retest reliability estimates range from *r* = 0.45 and *r* = 0.70. It correlates with other self-report depression measures like the BDI (Segal et al., 2008), and interviewer ratings of depression (Radloff, 1977).

#### Satisfaction with life questionnaire (SWL)

The Satisfaction with Life Questionnaire is a five-item scale designed to measure life satisfaction or subjective well-being. The internal consistency of this measure is good (α = 0.87) (Neto, 1993), with a 2 months test-retest reliability of *r* = 0.82, and a test-retest reliability of *r* = 0.50 over 10 weeks, and *r* = 0.54 over 4 years (Pavot and Denier, 1993).

#### Positive affect negative affect scale (PANAS)

The PANAS is a twenty-item instrument measuring two constructs, positive and negative affect, with two ten-item lists of words. The internal consistency of the PANAS, Positive Affect (PAS) and Negative Affect (NAS) scales, estimated using Cronbach's α was 0.89 for the PAS, and 0.85 for the NAS (Crawford and Henry, 2004). At an 8-week interval, the test-retest reliability for PAS ranged from *r* = 0.47 to *r* = 0.68 (Watson et al., 1988).

#### Neuroticism-extroversion-openness inventory- five factor inventory (NEO-FFI)

The NEO-FFI is a sixty-item personality questionnaire designed for use with adult individuals without overt psychopathology. It measures five personality factors: Openness to Experience, Conscientiousness, Extraversion, Agreeableness, and Neuroticism. The internal consistency of each of the factors is good, ranging from α = 0.76 to α = 0.88 (Sacier, 1998).

#### Procedure

*Appointment 1:* On Day 1, the experimenter greeted the participants, obtained informed consent, and then handed them written directions that described when and how to access the *Diary NPV website*, which contained a self-report NPV daily diary described above and the *Individual Differences website*, which contained the NPV questions described above.

The experimenter then verbally summarized when and how to access the two websites. Upon completion, the experimenter asked if the participants understood those directions. If a participant indicated she/he did not understand the directions, the experimenter rephrased those directions and continued to do so until the participant indicated complete understanding.

The experimenter then handed each participant a GPS device and verbally instructed him/her to carry the device on their person, preferably in a pocket, short of that in a backpack or purse, over the next 5 days. The participant was reminded to return on the pre-scheduled Appointment 2 date for a battery change, and then dismissed. At 6 p.m. on Day 1, a computer program emailed each participant a copy of instructions on how access and interact with the websites.

*Appointment 2:* If the participant's first appointment was on a Wednesday, Appointment 2 was on a Friday (Day 3). If the participant's first appointment was on a Friday, Appointment 2 was on the following Monday (Day 4). During Appointment 2, the participants returned to the laboratory, the experimenter changed the GPS batteries, and then dismissed the participants.

The computer program sent participants reminder emails on the address of the websites and the proper time to enter their data at 6 p.m. on each of Days 2–5.

*Appointment 3:* On Day 6 (Monday for participants who started on a Wednesday and Wednesday for participants who started on a Friday), before the participants arrived, the experimenter checked to ensure each participant had completed the self-report website tasks. If a participant had not completed the task, then the experimenter noted the fact, prepared the website, and after collecting the GPS device, instructed that individual to complete the web-based tasks.

Upon arrival, the GPS devices were recovered. Participants completed the Google NPV task described below. Those participants who had not completed all of the web-based tasks were asked to complete the tasks before moving on to the Google NPV task (described in the Materials and Measures section above).

After completing the Google NPV task, the experimenter offered the participants the opportunity to remove any data the GPS device may have recorded. None took advantage of this opportunity. The experimenter then debriefed the participants, awarded experimental credits, and dismissed them.

### GPS NPV analysis

The GPS software, provided with the devices, provided an “activity report” detailing paths travelled and places where participants stopped moving. The program defined a stop as a user-defined time of no detectable change in location. The present study defined a 10-minute interval as a stop. The program also provided time arrived, time departed, estimated address, distance between stops, and duration of stop for each stop. We used data from the activity report as a first estimate of NPV. Each individual stop was examined by clicking on a Google Maps link to visually inspect the possible *place* the participant visited.

We used the following rules to classify a stop as a *place* or not a *place* for the NPV estimate. First, if two consecutive stops had the same estimated address, then we scrutinized the two stops further. In this case, if the stops happened at two separate buildings, then both the first and second stop counted as places. If, however, the second stop was at the same building and/or within the confines of a fence or yard surrounding it, or if it looked like a single residential address, then the second stop did not count as a place. If the software reported two consecutive stops at an identical location, the two stops were treated as a visit to one place. If a stop happened at an intersection and it was obvious that the person was in transit, then it did not count as a place. Once we defined, counted, and entered place stops into a spread sheet, then that number served as an initial NPV count for that day.

We then plotted each individual's route and superimposed it on a map. Using the second-by-second speed estimates recorded by the GPS devices we screened for all reported speeds in excess of 75 miles per hour that went in apparently random directions (e.g., lack of roads), which we categorized as an anomaly. When an anomaly was detected, the place stops that came from the GPS software activity reports were plotted over the route map and, if one of the stops was due to an anomaly, the data point was removed from the NPV estimate. Anomalies did not ordinarily have a stop lasting longer than 10 min and, therefore, rarely ever was reported as a stop in the GPS software's activity report. We then added the appropriate NPV estimates to create a four day GPS NPV, a weekend GPS NPV, and a weekday GPS NPV variable.

*Convergent Validity:* To examine convergent validity we did two separate analyses. First, we examined the correlations among the two self-report measures of NPV and the GPS-based measure of NPV across the full 4 days. Second, because we expected the number and type of places visited to vary as a function of weekday versus weekend, we split and coded the data as weekday or weekend. We calculated the correlations among the two self-report based measures and the GPS-based measures of NPV for the full 4 days, the weekdays, and the weekends separately. We then used those correlations as indicators of convergent validity. This provided an initial examination of the weekend and weekday differential accuracy hypothesis outlined in the introduction.

*Nomological validity:* To examine nomological validity, we first constructed a measurement model comprised of a factor composed of variables related to an AL and a factor composed of variables related to a SL. We used a Split-Plot General Linear Model (GLM) to examine the relations among our Diary-, Google-, and GPS-based measures of NPV, AL and SL (as described below).

Data from the BDI, CES-D, SWL, Neuroticism (N), and Negative Affect (NA) scales comprised the SL factor. We estimated the SL factor by calculating a unit-weighted factor score. We first reverse scored the SWL, then standardized all the variables, and took their unit-weighted average (Gorsuch, 1983).

We estimated the AL factor the same way, using five measured variables, Positive Affect (PA), Life History (MK), Conscientiousness (C) Extraversion (E), and Agreeableness (A). We omitted Openness to Experience for two reasons: to keep AL and the SL factors as comparable as possible by including the same number of indicators, and, of the six variables that could have gone into AL, Openness to Experience fit AL less well, as assessed by the correlation of the individual indicator score to the AL composite factor score.

The Split-Plot GLM examined convergent and nomological validity of these measures. The outcome variable was composed of each of the three methods used to measure NPV. In other words, each person had six (or fewer due to missing data) values for NPV, one for Diary NPV, another for Google, and another for GPS on the weekdays, and yet another set of these three on the weekends. In total there were 369 NPV observations. Convergent validity was examined using orthogonal contrasts to compare Diary NPV, Google NPV, and GPS NPV. The first contrast (C1) compared the two self-report methods directly. The second contrast (C2) compared the average of the two self-report methods against the GPS directly. A dummy variable “Time” distinguished weekends and weekday effects.

The same analysis continued by including SL and AL as *predictors* of NPV as measured by the Diary-, Google-, and GPS-based methods. This, along with several interaction terms, tested several alternative hypotheses concerning convergent and nomological validity of the Diary, Google, and most importantly GPS measures of NPV (see Tables 1 and 2).

**Table 1:** **Nomological validity hypotheses tested by the interaction terms**.

**Predicted interactions**	**Hypotheses**
AL × SL	The number of places visited varies as a function of AL *and* SL
AL × Time	People high in AL visit more places on the weekend than the weekday
SL × Time	People high in SL visit fewer places on the weekend than the weekday
AL × SL × Time	Number of places visited differs on the weekend and weekdays differ as a function of AL *and* SL

This table is a list of interactional hypotheses which are entered in both Model 1 and Model 2 which test nomological validity. A statistically significant interaction term supports the prediction.

**Table 2:** **Convergent validity hypotheses tested by the interaction terms**.

**Predicted interactions**	**Hypotheses**
Time × C1	The accuracy of self-report NPV varies as a function of weekend vs. Weekday
Time × C2	The accuracy self-report NPV relative to GPS NPV varies as a function of weekend vs. weekday
AL × C1	People high in AL self-report differently as a function of weekend vs. Weekday
SL × C1	People high in SL self-report differently as a function of weekend vs. Weekday
AL × SL × C1	The accuracy of the self-report NPV differs as a function of Al *and* SL
AL × C2	The accuracy of self-report NPV *and* GPS NPV differentially vary as a function of AL
SL × C2	The accuracy of self-report NPV *and* GPS NPV differentially vary as a function of SL
AL × SL × C2	The accuracy of self-report NPV *and* GPS NPV differentially vary as a function of SL and AL

This table is a list of interactional hypotheses which are entered in both Model 1 and Model 2 which test convergent validity. A statistically significant interaction term provides evidence against the prediction.

We tested two models, both of which predicted NPV. The first gave causal priority to AL (Equation 1) and the second gave causal priority to SL (Equation 2). We did so for two reasons. First, we did not have a theoretically grounded reason to give causal priority to either AL or SL. Second, because a Split-Plot GLM partitions variance hierarchically, that is, a Split-Plot GLM estimates the between-subjects variance before the within-subjects variance.

Hence, the order of AL and SL reversed the order of the two main predictor variables. If we did not do this, and the second term in model was not significant, a critic could legitimately argue that any statistical conclusion we reached was faulty. That is, our critic could allege that we falsely concluded that our measures lacked nomological validity because the conclusion is based on a statistical test designed to test competing causal hypotheses and not our proposed nomological net. The two predictor variables could be so closely related, due to a spurious or causal relationship, that the first variable in the model accounts for the majority of the systematic variance, leaving little or none for the second variable, but the cause of the multicolinearity is not addressed. Running the model both ways obviates this possibility.

We used the following equation to construct Model 1.

(1) $$\begin{array}{l} NPV=AL+SL+AL\times SL+SID+C1+C2+Time\times C1\\ \;+Time\times C2+AL\times Time+SL\times Time+AL\times SL\\ \;\times Time+AL\times C1+SL\times C1+AL\times SL\times C1+AL\\ \;\times C2+SL\times C2+AL\times SL\times C2+Time\times SID+C1\\ \;\times SID+C2\times SID \end{array}$$

We used the following equation to construct Model 2.

(2) $$\begin{array}{l} NPV=SL+AL+SL\times AL+SID+C1+C2+Time\times C1\\ \;+Time\times C2+SL\times Time+AL\times Time+SL\times AL\\ \;\times Time+SL\times C1+AL\times C1+SL\times AL\times C1+SL\\ \;\times C2+AL\times C2+SL\times AL\times C2+Time\times SID+C1\\ \;\times SID+C2\times SID \end{array}$$

The bolded elements in Equation (2) represent the critical differences (reversed causal priorities) between Equations (1) and (2). [The raw data, SAS code, and R code for all of the analyses described in this section and additional figures are available at http://web.uct.ac.za/depts/psychology/staff/people/pedro.html].

We then examined two causal interpretations of the data, the first based on Model 1 and the second based on Model 2. This permitted us to avoid biases in our interpretation of the statistical results. For example, if Model 1 demonstrated a statistically significant predictive relation between SL and NPV, the analysis would clearly indicate a relation between SL and NPV, independent of AL. If, however, Model 2 simultaneously demonstrated a statistically significant predictive relation between AL and NPV, then that analysis would clearly indicate a relation between AL and NPV, independent of SL. Taken together, the analyses would confirm that SL and AL independently predict NPV. Obviously several other patterns may emerge. For example, if Model 1 detected a statistical relation between AL and NPV but Model 2 detected no statistical relation between AL and NPV, we would conclude that, after controlling for SL, AL does not independently predict NPV. We will use a similar interpretive strategy in light of any other statistically revealed data patterns.

In summary, we used a bi-variate approach to establish one level of convergent validity. Following that, we considered the joint results of two separate Split-Plot General Linear Models to test the convergent and nomological validity of the GPS-based measure of NPV and to disambiguate alternative hypotheses designed to explain the how activity schedules or other factors influence or constrain activity throughout the week.

*Missing Data:* Due to the complexity of the study, there was a considerable amount of missing data. We know that much of the missing data occurred because of technical difficulties. For example, we lost data due to a website crash during November of 2010 and due to research assistants, not familiar with the GPS devices, accidentally erasing rather than downloading data sets from the GPS devices.

We therefore analysed our missing data. To quantify missing data we created a new data set and dichotomized all scores; a one was entered if there was a score for an individual measure and a zero if there was no score for that measure. We then conducted two separate inferential analyses. The first, tested if any of the measured demographics or individual differences variables predicted missing data, and the second tested patterns of missing data related to the day the task took place. This analysis investigated the Individual Differences Self-Report Questionnaires, Diary, and the GPS methods because these were the tasks that did not take place in the laboratory and were subject to non-compliance.

In general most people completed the Diary, Self-Report Questionnaires, and GPS tasks on time and completely. The majority of those who did not complete one of the tasks also did not complete the remaining tasks. Because we did not detect systematic relations among demographics variables, day of week variables, and missing data, we decided to use all available data from each participant; we did not impute missing data (See Wolf, 2011 for a detailed account of this analysis).

## Results

### Convergent validity

Table 3 provides the correlations among the NPV variables (*r*), significance values (*p*), number of participants (*N*), and 95% confidence intervals(CI) during the entire 4 days of the study. Each correlation (*r*) reached statistical significance, indicating that the data provided by the GPS-based measure converge on the data provided by the self-report based measures. Hence, these three methods appear to provide valid measures of the same construct (number of places visited).

**Table 3:** **Correlations among NPV variables**.

	**Google NPV**	**GPS NPV**	**Diary NPV**
		*r* = 0.37	*r* = 0.56
Google NPV	–	*p* = 0.004	*p* = 0.001
		*N* = 58	*N* = 48
		CI = 0.01, 0.49	CI = 0.35, 0.75
			*r* = 0.40
GPS NPV		–	*p* = 0.004
			*N* = 50
			CI = 0.06, 0.57
Diary NPV			–

Table 4 provides the correlations among the NPV variables (*r*), significance values (*p*), number of Ps (*N*), and 95% confidence intervals (CI) during the weekend and weekday. Each weekday correlation (*r*) reached statistical significance indicating convergent validity of our GPS on the weekdays. The same pattern of correlations are found on the weekends, however, one correlation (Diary NPV and GPS NPV) did not reach statistical significance, however, the magnitude of the correlation was in the same order as the others. The failure to reach statistical significance is likely related to the smaller sample size for that correlation. Both of these measures required active data collection from our participants and this resulted in more missing data. From this perspective, these three methods appear to provide equivalent measures of the same construct (number of places visited) independent of the day the data were collected.

**Table 4:** **Correlations among NPV weekend and weekday variables**.

	**Google NPV**	**GPS NPV**	**Diary NPV**
		***r* = 0.32**	***r* = 0.48**
Google NPV	–	***p* = 0.02**	***p* < 0.01**
		***N* = 52**	***N* = 46**
		**CI = 0.00, 0.51**	**CI = 0.21, 0.68**
	*r* = 0.32		***r* = 0.29**
GPS NPV	*p* = 0.02	–	***p* = 0.05**
	*N* = 58		***N* = 45**
	CI = −0.01, 0.43		**CI = 0.00, 0.48**
	*r* = 0.34	*r* = 0.28	
Diary NPV	*p* = 0.02	*p* < 0.05	
	*N* = 48	*N* = 50	–
	CI = 0.04, 0.56	CI = −0.06, 0.48	

The correlations (r), significance values (p), number of participants (N), and 95% confidence intervals (CI) among the weekend NPV variables are bolded and in the upper right hand corner of the table. The correlations (r), significance values (p), number of participants (N), and 95% confidence intervals (CI) among the weekday NPV variables are underlined and in the lower left hand corner of the table.

### Nomological validity

*Descriptive statistics for SL and AL:* Table 5 provides the means and standard deviations for the variables that composed the SL and AL factors used in this study (for further details see Wolf, 2011).

**Table 5:** **Descriptive statistics, possible ranges, and sample Cronbach's α for predictor variables of the sedentary lifestyle and active lifestyle factors**.

	**Mean**	**Standard deviation**	**Range**	**Possible range**	**Cronbach's α**
**SEDENTARY LIFESTYLE**					
CES-D	38.42	6.43	(0, 55)	(0, 60)	0.93
BDI	4.49	5.18	(0, 52)	(0, 63)	0.90
Negative affect	19.86	6.90	(10, 46)	(10, 50)	0.89
SWL	25.38	5.86	(5, 35)	(5, 35)	0.93
Neuroticism	−4.26	8.29	(−24, 15)	(−24, 24)	0.82
**ACTIVE LIFESTYLE**					
Conscientious	7.63	6.92	(−12, 18)	(−24, 24)	0.85
Extraversion	8.34	6.81	(−16, 24)	(−24, 24)	0.80
Agreeableness	8.43	5.98	(−11, 12)	(−24, 24)	0.72
Mini-K	1.22	0.70	(−1, 2.8)	(−3, 3)	0.76
Positive affect	36.81	7.06	(14, 50)	(10, 50)	0.93

*Descriptive statistics for outcome variable:* Table 5 provides the means and standard deviations for the NPV variables as obtained through the Google-, Diary-, and GPS-based methods. As can be seen in Table 6, the overall NPV estimates for each of the three methods appear to be equivalent, and the equivalency of these means are confirmed later in the paper in the Split-Plot GLM section of the data analysis. On average, the participants visited between 22 and 24 places across 4 days, depending on the method of measurement. The standard deviation for the same times range between 9.2 and 12.7. The methods also produce similar estimates for the weekend (between 9.3 and 10.75 NPV) and weekdays (between 13.39 and 13.54). It appears however that the participants visited, on the average, about 3 *fewer* places on the weekend than the weekdays.

**Table 6:** **Descriptive statistics of the outcome variable**.

**Variable**	**Mean**	**Standard deviation**
**OVERALL NPV**		
Google NPV	23.00	10.10
Diary NPV	23.94	12.47
GPS NPV	22.26	9.22
**WEEKEND NPV**		
Google NPV	9.90	5.14
Diary NPV	10.75	6.26
GPS NPV	9.30	5.65
**WEEKDAY NPV**		
Google NPV	13.54	6.13
Diary NPV	13.39	7.96
GPS NPV	13.77	5.09

### Constructing SL and AL factors

*SL:* We constructed the SL factor using the theoretically specified individual indicators as described in the statistical analysis section above. Table 7 provides the correlations between SL and the individual indicators. These correlations range between *r* = 0.61 and *r* = 0.91 indicating that each of these measures can be considered legitimate indicators of a single higher order factor.

**Table 7:** **Correlations between active lifestyle (AL) and sedentary lifestyle (SL) factors and all indicator variables**.

**Variables**	**AL**	**SL**
AL	*r* = 1.00	*r* = −0.30
		*p* = 0.01
	*n* = 84	*n* = 84
SL	*r* = −0.30	*r* = 1.00
	*p* = 0.01	
	*n* = 84	*n* = 87
MK	*r* = 0.67	*r* = −0.09
	*p* < 0.01	*p* = 0.47
	*n* = 79	*n* = 75
C	*r* = 0.74	*r* = −0.17
	*p* < 0.01	*p* = 0.47
	*n* = 81	*n* = 80
E	*r* = 0.78	*r* = −0.34
	*p* < 0.01	*p* < 0.01
	*n* = 82	*n* = 79
A	*r* = 0.59	*r* = −0.30
	*p* < 0.01	*p* = 0.01
	*n* = 81	*n* = 80
PA	*r* = 0.77	*r* = −0.15
	*p* < 0.01	*p* = 0.18
	*n* = 78	*n* = 81
BDI	*r* = −0.25	*r* = 0.91
	*p* = 0.03	*p* < 0.01
	*n* =	*n* = 75
NAS	*r* = −0.3	*r* = 0.82
	*p* = 0.81	*p* < 0.01
	*n* =	*n* = 80
DWL	*r* = −0.49	*r* = 0.61
	*p* < 0.01	*p* < 0.01
	*n* =	*n* = 89
N	*r* = −0.34	*r* = 0.83
	*p* < 0.01	*p* < 0.01
	*n* =	*n* = 80
CESD	*r* = −0.02	*r* = 0.72
	*p* = 0.86	*p* < 0.01
	*n* =	*n* = 81
AL	*r* = 1.00	*r* = −0.30
		*p* = 0.01
	*n* = 84	*n* = 84
SL	*r* = −0.30	*r* = 1.00
	*p* = 0.01	
	*n* = 84	*n* = 87

*AL:* We constructed the AL factor using the theoretically specified individual indicators described in the statistical analysis section above. Table 7 provides the correlations between AL and the individual indicators. These correlations range between *r* = 0.59 and *r* = 0.78 indicating that each of these measures can be considered legitimate indicators of a single higher order factor.

Both AL and SL correlated with each other (*r* = −0.30) and for the most part indicators of the SL factor do not correlate with the AL factor and vice versa much higher than the *r*–0.30 correlation between the two factors. The only exception being the DWL with a correlation of −0.49.

### GLM approach: convergent and nomologicalvalidity analyses

*Model 1* used a Split-Plot GLM to examine convergent and nomological validity. To test nomological validity, the model used SL and AL as predictors of NPV as measured by the Diary-, Google-, and GPS-based methods. To test convergent validity, the model compared Diary NPV, Google NPV, and GPS NPV. The model also tested several of the specific predictions outlined in Table 1 by analysing theoretically specified interaction terms.

The model was specified by entering the between subjects variables in this order, AL, SL, the interaction term AL × SL, and a subject identifier (SID). These variables were followed with the within subject variables, weekday vs. weekend (Time), the contrast codes C2 and C1 (C2 compared the two self-report NPVs against GPS NPV, and C1 compared the two self-report NPV measures). After entering these variables we entered this series of theoretically specified interaction terms Time × C2, Time × C1, Time × AL, Time × SL, Time × AL × SL, AL × C2, S × C2, AL × SL × C2, AL × C1, SL × C1, AL × SL × C1. For a rationale of each of these interaction terms see Table 1. Finally, the following error terms were entered into the model, Time × SID, C2 × SID, and C1 × SID.

Table 8 provides the between- and within-subjects sum of squares, the name of the predictor, the *F*-value (ratio of explained over unexplained variance for that variable), degrees of freedom, the probability of obtaining the associated *F*-value when assuming the null hypothesis is true, the semi-partial *R*^2^ for both between and within-subjects components of variance, each variables' partial *R*^2^, the model's *R*^2^, and the full model's F table.

**Table 8:** **Hypothesis tests and effect sizes for between-subjects and within-subjects predictors**.

**Type**	**Predictor**	**NDF, DDF**	***F*-value**	***p*-value**	**Semi-partial *R*^2^**	**Partial *R*^2^**
BS	AL	1, 63	12.23	0.00	0.068	0.151
BS	SL	1, 63	0.18	0.67	0.001	0.002
BS	AL × SL	1, 63	0.23	0.64	0.001	0.003
BS	SID	63, 87	4.17	0.00	0.381	0.844
BS	Total	–	−	−	0.451	1.00
WS	Time	1, 61	45.87	0.00	0.072	0.176
WS	C2	1, 51	1.51	0.22	0.008	0.019
WS	C1	1, 51	3.23	0.08	0.011	0.027
WS	Time × C2	1, 87	0.93	0.37	0.001	0.004
WS	Time × C1	1, 87	0.35	0.56	0.002	0.001
WS	Time × AL	1, 61	0.10	0.75	0.001	0.001
WS	Time × SL	1, 61	0.82	0.37	0.000	0.003
WS	Time × AL × SL	1, 61	1.44	0.24	0.001	0.005
WS	AL × C2	1, 51	0.35	0.56	0.002	0.003
WS	SL × C2	1, 51	0.18	0.67	0.001	0.000
WS	AL × S × C2	1, 51	0.04	0.84	0.002	0.000
WS	AL × C1	1, 38	0.62	0.44	0.001	0.006
WS	SL × C1	1, 38	0.01	0.92	0.000	0.000
WS	AL × S × C1	1, 38	0.03	0.85	0.000	0.002
WS	Time × SID	61, 87	1.16	0.26	0.105	0.255
WS	C2 × SID	51, 87	1.93	0.00	0.108	0.263
WS	C1 × SID	38, 87	1.83	0.01	0.098	0.235
WS	Total	–	−	−	0.413	1.00
Summary	Model	221, 302	2.47	0.00	0.864	0.864
	Error				0.136	0.136
	Total				1.00	1.00

The acronyms used in this table are as follows: Numerator Degrees of Freedom (NDF), Denominator Degrees of Freedom (DDF), Degrees of Freedom (DF), Between Subjects (BS), and Within Subjects (WS). We calculated the semi-partial R^2^ for each BS and WS variables by separately adding up the sum of squares for all the BS variables and then adding up the sum of squares for the WS variables. These values served as our BS and WS total sum of squares. Using these values, we then calculated the semi-partial R^2^ for each BS variable by dividing each BS variable's sum of squares by the BS total sum of squares. Finally, we calculated the semi-partial R^2^ for the WS variables by dividing each WS variable's sum of squares by the WS total sum of squares.

AL and Time significantly predicted NPV. The other predictors of interest regarding nomological validity (e.g., SL, SL × Time etc.) did not. The remaining terms, SID, C2 × SID, and C1 × SID, which were significant error terms, indicated that individuals differ in the number of places visited, how they self-report NPV, and that self-report- and GPS- based NPV differed for some individuals. These error terms are of no theoretical interest in the present context.

Hence Model 1 indicates that AL and Time are statistically significant and SL is not statistically significant. When it comes to the between-subjects variance component, AL accounted for around 15% of the variance, and the majority (84%) of the variance was accounted for by the SID variable, which is an estimate of the unexplained variance among individuals. When it comes to the within subjects variance, the variable Time accounted for 18% of that variance and the majority of the rest of the variance (75%) was accounted for by the interactions of SID with Time, C2, and C1. This indicates that a substantial portion of the unexplained variance is due to individual differences in the magnitude of the method effects and time of week effects. Importantly, the main effects of C1 and C2 only accounted for 5% of the total within subjects variance.

Based on the semi-partial *R*^2^, AL and Time accounted for approximately 7% of the total variance in the outcome variable. Together, each of our contrast codes accounted for about 2% of the total variance indicating how small the method effect was which strengthens our convergent validity hypothesis.

*Model 2:* In an attempt to disconfirm the conclusion that AL and Time but not SL are of theoretical interest in this context, we ran an identical analysis but entered SL before AL throughout. This produced Model 2. This analysis provided a check against alternative interpretations of these data as offered by our hypothetical critic or a misguided reviewer.

Table 9 provides the between- and within-subjects sum of squares, the name of the predictor, the *F*-value indicating the status of the predictor, degrees of freedom, the probability associated with the *F*-value, the semi-partial *R*^2^ for both between and within-subjects components of variance, each variables' partial *R*^2^, the model's *R*^2^, and the full model's F table. As before, the table indicates that AL and Time are significant predictors of NPV, but the other predictors of interest were not. Importantly, there was no detectable relation between SL and NPV, indicating the idea that the AL and SL variables are so closely related that AL (in Model 1) accounted for the majority of the systematic variance, leaving little or none for the SL variable is in error. This result indicates that the SL is not an important part of the nomological net.

**Table 9:** **Hypothesis tests and effect sizes for between-subjects and within-subjects predictors**.

**Type**	**Predictor**	***NDF, DDF***	***F*-value**	***p*-value**	**Semi-Partial *R*^2^**	**Partial *R*^2^**
BS	SL	1, 63	0.13	0.72	0.002	0.00
BS	AL	1, 63	12.29	0.00	0.068	0.15
BS	AL × SL	1, 63	0.23	0.64	0.001	0.00
BS	SID	63, 87	4.22	0.00	0.383	0.84
BS	Total	–	–	–	0.454	1.00
WS	Time	1, 61	45.87	0.00	0.073	0.18
WS	C2	1,51	1.51	0.22	0.008	0.02
WS	C1	1,51	3.14	0.09	0.011	0.03
WS	Time × C2	1, 87	0.94	0.33	0.002	0.00
WS	Time × C1	1, 87	0.35	0.55	0.001	0.00
WS	Time × SL	1, 61	0.66	0.42	0.001	0.00
WS	Time × AL	1, 61	0.26	0.61	0.001	0.00
WS	Time × AL × SL	1, 61	1.44	0.23	0.002	0.00
WS	S × C2	1, 51	0.28	0.60	0.000	0.00
WS	A × C2	1, 51	0.26	0.62	0.000	0.00
WS	AL × SL × C2	1, 51	0.04	0.84	0.000	0.00
WS	SL × C1	1, 38	0.06	0.81	0.000	0.00
WS	AL × C1	1, 38	0.55	0.46	0.000	0.00
WS	AL × SL × C1	1, 38	0.03	0.85	0.000	0.00
WS	Time × SID	61, 87	1.17	0.24	0.105	0.25
WS	C2 × SID	51, 87	1.95	0.00	0.109	0.26
WS	C1 × SID	38, 87	1.90	0.01	0.097	0.24
WS	Total	–	–	–	0.411	1.00
Summary	Model	221, 302	2.47	0.00	0.864	0.864
	Error				0.136	0.136
	Total				1.00	1.00

The acronyms used in this table are as follows: Numerator Degrees of Freedom (NDF), Denominator Degrees of Freedom (DDF), Degrees of Freedom (DF), Between Subjects (BS), and Within Subjects (WS). We calculated the semi-partial R^2^ for each BS and WS variables by separately adding up the sum of squares for all the BS variables and then adding up the sum of squares for the WS variables. These values served as our BS and WS total sum of squares. Using these values, we then calculated the semi-partial R^2^ for each BS variable by dividing each BS variable's sum of squares by the BS total sum of squares. Finally, we calculated the semi-partial R^2^ for the WS variables by dividing each WS variable's sum of squares by the WS total sum of squares.

Again, the error terms, SID, C2 × SID, and C1 × SID, were significant predictors but are of no theoretical interest in the present context. We found the same pattern of partial and semi-partial *R*^2^ estimates in Model 2 as we did in Model 1.

### Parameter estimates

Although not central to our major purpose, we ran a final regression analysis to refine our estimates of the relations between the significant predictors, AL and Time, and our criterion variable NPV (Cohen and Cohen, 1983). Table 10 provides unstandardized parameter estimates and standard errors for the intercept, AL, and Time. These estimates indicate that on average, people visit 11.87 places on any given 2 days. The model also indicates that for every increase in a standardized AL unit, a person visits 1.79 more places; conversely, for every decrease in a standardized AL unit, a person visits 1.79 fewer places. The parameter estimate Time indicates that, on average, a person visits 3.68 fewer places during a weekend. These estimates of course, apply only to the population that our sample represents. Clearly, these estimates do not represent populations such as human infants, the elderly, or people with professions that require travel or that work at home.

**Table 10:** **Parameter estimates for the significant variables**.

**Predictor**	**Parameter estimate**	**Standard error**
Intercept	11.87	0.36
AL	1.79	0.36
Time	−3.68	0.53

## Discussion

The significant correlations among NPV measures- weekday, weekend, and the entire four day period clearly indicate that Diary-, Google Maps-, and GPS-NPV measure the same construct and support the convergent validity of the measures at some level. The only non-significant correlation was the weekend Diary and GPS-NPV correlation with an exact *p*-value of 0.054. Despite the statistical significance of most of these correlations, the magnitude of the correlations are relatively weak given that they are supposed to measure the same behavior. The results of the second analysis, indicating non-significant mean differences in NPV recorded by the three measures also support this conclusion.

None of the predicted interaction that reflected possible alternatives to convergent validity reached statistical significance and accounted for a negligible amount of variance. Although interactions can be difficult to detect statistically (McClelland and Judd, 1993), the large number of NPV observations (*n* = 369) ensured sufficient power to disconfirm these alternatives. Even if there are relations between some or all of these interaction terms and NPV, which require additional statistical power to detect, those relations are too small to be of practical or, for now, theoretical interest.

AL is a statistically significant predictor of NPV no matter how NPV was measured. This relationship is consistent with what has already been described in the literature (e.g., Rhodes and Smith, 2006; Mata et al., 2012). *Ceteris paribus*, happy people are active people. This evidence supports the nomological validity of the NPV measures.

Surprisingly, predictions in the literature linking SL and NPV were not supported. This lack of evidence could be due to several limitations of our study, the most likely being that our sample did not include clinically depressed individuals. In the literature, the relation between negative affective states and physical activity is usually reported when the samples include pathologically depressed individuals. It is therefore possible a restricted range related to negative affect in our sample of healthy college students prevented the detection of this relation. Another possibility is that the factor structure of this theoretically specified factor has not been empirically demonstrated either by us or in the literature. A more extensive study, including many more participants, would need to be conducted in order to establish the factor structure of this construct.

### Cautionary notes

We made the conditions for self-reporting NPV as ideal as possible. We carefully constructed each question, we ensured that the behaviors were self-reported on the same day they occurred and we sent email reminders, or provided maps as cues to their behavior when they had to remember past 1 day to obtain these results. Hence, we caution, the strength of the relations that suggest convergent validity may be even less robust under less stringent conditions.

The accuracy of self-reported behavior may decline or participants may fail to carry the GPS devices under less tightly controlled conditions. On the face of it, one may think that an obvious data pattern occurs when participants do not carry the devices– a data pattern that allows the researcher to identify a source of measurement error and rectify the problem. The participants appear to stay in one place over an extended period of time (e.g., 24 h). One must exercise caution, however, when making this inference. One of our irrecoverably de-identified participants, for example, claimed to have spent the whole weekend smoking marijuana and playing video games at home. The GPS device recorded no movement at all. That data pattern is identical to data patterns recorded when the individual leaves the device at home. Disambiguating this data pattern, without supporting self-report or other technology (e.g., accelerometers) is problematic.

There is as yet an unremarked feature of these data most clearly shown in Tables 3 and 4. A brief examination of these tables reveals correlations among the self-report and GPS measures of NPV range from 0.28 and 0.37. The correlations between the self-report based NPV measures are stronger, ranging between 0.34 and 0.56, permitting us to infer that shared method variance produces these higher correlations. What these methods share is that the participants must *remember* to report NPV via the Google and Diary methods. Although these relations are statistically significant, they indicate that a great deal of variance remains to be explained.

This sets a puzzle, how can these correlations be so low, but the mean NPV's across the three methods be statistically indistinguishable. The lack of mean differences indicates that there are no systematic biases originating from the different methods. One answer to this puzzle is that the low correlations suffer because of no small amount of measurement error, and that the use of multi-method measurement should is important. Determining the sources of these errors will require different designs and, perhaps, more sophisticated statistical approaches to the data obtained from those designs.

In summary, we have demonstrated convergent validity among three measures of daily activity using two distinct analytical approaches. We have also demonstrated the nomological validity of the new measure using two GLM based models. Both convergent and nomological validity belong to the more general category Construct Validity (Campbell, 1960). Because the GPS NPV convergent on both self-report based measures of NPV and were predicted by at least one indicator of a nomological net, we can say with some confidence a GPS device, carried faithfully, provides an adequate measure of the number of places an individual visits and with additional analyses can provide a host of other measures of daily weekly or monthly activity (see Wolf and Jacobs, 2010; Wolf, 2011; Miller, 2012).

### Conflict of interest statement

The authors declare that the research was conducted in the absence of any commercial or financial relationships that could be construed as a potential conflict of interest.
